# Social Distancing in Tourism Destination Management during the COVID-19 Pandemic in China: A Moderated Mediation Model

**DOI:** 10.3390/ijerph182111223

**Published:** 2021-10-26

**Authors:** Hui Zhang, Min Zhuang, Yihan Cao, Jingxian Pan, Xiaowan Zhang, Jie Zhang, Honglei Zhang

**Affiliations:** School of Geographic and Oceanographic Sciences, Nanjing University, Nanjing 210023, China; zhhui93@outlook.com (H.Z.); minzhuang521@smail.nju.edu.cn (M.Z.); caoyh7@smail.nju.edu.cn (Y.C.); panjingxian0827@163.com (J.P.); cuxz2323@126.com (X.Z.); jiezhang@nju.edu.cn (J.Z.)

**Keywords:** protection motivation theory, COVID-19, protective behaviors, social distancing, destination management

## Abstract

While protective measures in response to infectious diseases may reduce the freedom of tourists (regarding their behaviors), few studies have documented the effects of destination protective measures on the self-protective behaviors of tourists. By applying the protection motivation theory, this study examines the effects of perceived destination protective supports on the social distancing intentions of tourists during the COVID-19 pandemic. The results reveal significant relationships among perceived destination support, coping appraisal, threat appraisal, and the social distancing intentions of tourists. Moreover, two cognitive appraisals—toward the pandemic—partially mediate the relationship between perceived destination support and social distancing intention, and this mediational process is ‘intervened’ with by social norms. This has implications on whether tourist destinations apply more rigorous social distancing polices during the COVID-19 pandemic, to enhance the coping confidence behaviors of tourists, without causing anxiety and fear, and to achieve the goal of enhancing tourists’ intentions to protect themselves.

## 1. Introduction

In December 2019, a novel coronavirus (COVID-19) appeared in Wuhan, China, and rapidly spread throughout the world. As of March 16, 2020, the cumulative number of confirmed patients worldwide exceeded 167,000, with more than 86,000 cases confirmed outside of China [1]. Protective behaviors in response to infectious diseases will have a considerable impact on the pandemic process [2]. Some important health protective behaviors can manifest in social distancing or via a reduction in social contact between individuals, in response to the presence of disease [3]. This is particularly useful during the COVID-19 pandemic when community transmission is believed to have occurred [4]. During the COVID-19 pandemic, the Centers for Disease Control and Prevention (CDC) revised the definition of social distancing as “keeping a safe space between yourself and other people who are not from your household, and maintaining distance (at least six feet or arms’ length) from others when possible [5].” To slow the spread of the pandemic when going out in public, it is important to stay at least six feet away from other people and wear a mask [5]; these behaviors are quickly becoming the new normal in many countries. To avoid exceeding critical care capacities, some researchers found that prolonged or intermittent social distancing might be necessary until 2022 [6]. After the enactment of strict government policies during the Chinese Lunar New Year holiday (e.g., encouraging people to stay at home; canceling public events; and closing scenic spots, schools, libraries, museums, and factories), Chinese citizens employed protective behaviors to defend themselves against COVID-19. They stayed at home, limited social contact, and wore masks when they needed to visit public places. These behaviors can be defined as “social distancing” [7]. This combination of social distancing and a range of strict pandemic control actions have prevented new infections in China, which have been declining since March 2020.

As domestic travel and work slowly resume in the country, the Chinese government started to remove inter-city travel restrictions in most low-risk areas, especially during the May Day holiday in 2020. However, to ensure the safety of the general public, and reduce the risk of spreading COVID 19, the Chinese CDC implemented a few key policies on travel health during the May Day holiday on April 30. Most were related to social distancing; for example, keeping at least one-meter distance when playing, wearing masks indoors and in crowded places, reducing the use of public transportation, opting for take-out meals, and sitting at the same side of the table to avoid face-to-face contact [8]. Using the opportunity brought about by explosive domestic travel during the COVID-19 outbreak in China, the entire tourism destination ecosystem might need to be reimagined and re-engineered. Although governments worldwide have already introduced various social distancing measures, personal compliance is essential [9]. However, these measures entail considerable restrictions to individual freedom [10], which require consideration by both tourists and destination managers. Therefore, the support of tourism destination managers toward social distancing during COVID-19 should be comprehensively discussed.

Research on the self-protective behaviors of tourists has been divided into two main streams: exploring self-protection behaviors against destination risk [11,12] and estimating impacts on the future travel behaviors [13,14,15]. Researchers found that government policies could be effective interventions that improve post-disaster tourism recovery [16]. During the COVID-19 pandemic, social distancing behaviors require not only destination guidance [9], but also personal compliance and self-efficacy from tourists. Although a series of specific crises and pandemic diseases have been studied [17,18,19], most focused on the destination risk perception; few studies have documented the effects of destination protective measures on the protective behavioral intentions of tourists. Moreover, China was the first country to experience the COVID-19 outbreak and the first to reduce its cases significantly [20].

To fill this knowledge gap, we measured the effects of destination guidance on social distancing intentions of tourists and explored the intrinsic dynamics and cognition processes of the social distancing behaviors of tourists in China. Among a wide variety of models to investigate the health behavior of individuals (e.g., health belief model, social cognition theory, and the theory of planned behavior) [21,22], the protection motivation theory (PMT) is one of the most comprehensive models used empirically. It explains how people cognitively assess a particular threat and perform protective behaviors [23]. Initially, PMT was developed based on expectancy-value theory [24], and revised to include inputs to the model and two cognitive mediating processes [23]. Input to the model includes two sources of information (environmental source and intrapersonal source), and the cognitive mediational process includes an individual’s threat assessment (threat appraisal) and perceived efficacy in coping (coping appraisal), which evokes protection motivation and behavior; some also include the input process, including environmental sources of information and intrapersonal sources of information [23]. Regarding the detailed cognitive appraisals, PMT is widely used in the field of public health [25], including disease prevention, healthy lifestyles, and self-protective or pro-environmental behaviors [26]. Some researchers have used PMT to examine the intentions of self-protective behaviors during a hypothetical pandemic [3,27]. PMT is also useful in explaining the behavioral dimension of travel attitudes, which can provide an interdisciplinary methodology to study tourism through a health science approach [28], especially regarding the self-protective behaviors of tourists against health risk situations [11].

Based on PMT, we established a theoretical framework to explore the relationships between perceived destination support, the cognitive mediating process, and social distancing behaviors in a public health-related tourism context. The key questions are: (1) will the perceived destination support, regarding social distancing measures, evoke the threat assessment of traveling during the pandemic period? (2) Will perceived destination support affect the coping response of tourists? (3) How does perceived destination support affect the tourists’ self-protective behaviors, with cognitive appraisal? (4) Considering the disagreements that may arise in joint actions under different personalities and national social environments—how can social norms affect the PMT model? This study explored the antecedents and behavioral consequences of tourists’ cognitive appraisals after the pandemic outbreak. It contributes to the literature in the following ways: first, it tests whether the overcrowding perception can be organized into threat appraisal during the pandemic period. Second, it examines the multi-variate relationships between the perceived destination support of protection measures and tourists’ social distancing intentions with two independent cognitive mediating processes: threat appraisal and coping appraisal. The results of this analysis have important theoretical implications for the studies of epidemic travel behaviors. Third, by examining the moderating effect of tourists’ social norms, this study provides new insights into how the effect of perceived destination support on social distancing intention with the mediation process of two appraisals is strengthened by those with high-level social norms. Finally, this study discusses the cognitive process of tourists, for the destination authority, during a specific period. The results are expected to help researchers understand the cognitive process of the social distancing behaviors of tourists in China and provide meaningful insights and suggestions for other countries. Thus, the findings from our study can help practitioners recognize tourist management and risk mitigation during the pandemic, and accelerate tourism recovery post-pandemic.

## 2. Literature Review and Hypotheses Development

### 2.1. Perceived Destination Support as the Input

**Figure 1:**
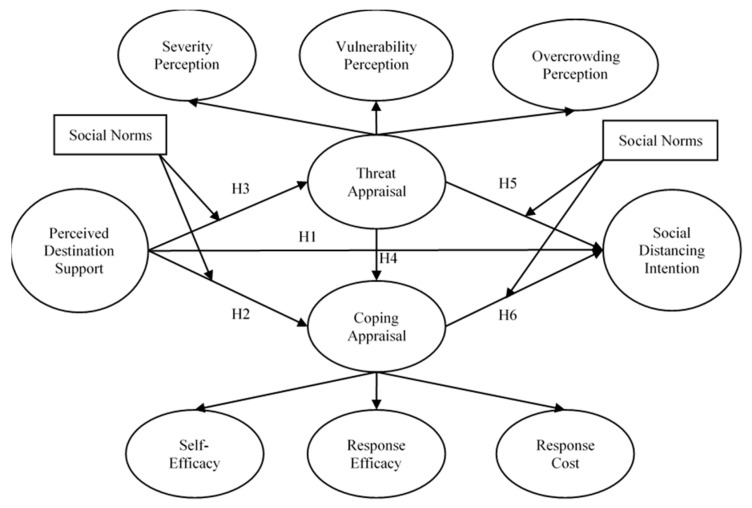
The proposed model. Based on the PMT and the research context.

The stimulus that induces threat appraisal and coping appraisal was often related to protective behaviors according to PMT, and inputs to the model include environmental sources of information and intrapersonal sources (Figure 1). As this theory has been successfully used in a variety of tourism studies, tourists’ personal factors and their perceptions of destinations can be the sources of information. Personal factors include tourists’ previous knowledge [29], habits [30], and convenience orientation [31]. The perception of destination includes both the perceived destination risks [13,32], and perceived destination benefits [31]. Destination governments and companies often launch various initiatives and support structures in regard to the prevention and control of crisis events [33], such as encouraging people to avoid mass gatherings, constructing online services, control measures to ensure physical distancing, and supervisory networks of COVID-19 information during the pandemic [8].

Perceived destination support can be the individual’s consideration of the effectiveness of destination support (in coping with risk); it could have a positive and significant impact on the protective behavioral intentions of tourists [18]. Regarding perceived destination benefits, perceived destination support could include the input of a PMT model; the relationship between perceived support and protective behavior intention is mediated by coping appraisal [34] and perceived threat [35]. Based on the relationship between perceived destination support and the PMT model, the following hypotheses are proposed:

**Hypothesis** **1** **(H1).** *Perceived destination support has a positive effect on tourists’ social distancing intentions.*

**Hypothesis** **2** **(H2).** *Perceived destination support has a positive effect on tourists’ threat appraisals.*

**Hypothesis** **3** **(H3).** *Perceived destination support has a positive effect on tourists’ coping appraisals.*

### 2.2. The Cognitive Mediating Process of PMT

Threat appraisal and coping appraisal are key adaptation factors in the cognitive mediating process of PMT [23]. Threat appraisal is primarily a combination of the individual’s judgment of the probability of threat occurrence (vulnerability perception) and the noxiousness of a threat in a given event (severity perception) [24,25]. Moreover, it indicates how effective the recommended action would be in response to the specific threat [11]. Therefore, during COVID-19 pandemic, threat appraisal refers to tourists’ severity perception and vulnerability perception against virus, but also can include other psychological trade-offs of the associated threat. Considering the particularity of community transmission and tourism mobility, we speculate that overcrowding risk will be the most considerable associated threat during official holidays [36]. Overcrowding perception as an independent dimension, is not a single psychological feeling, but is complex and influenced by user conflict as well as by unwanted visitor behaviors and resource conditions [37]. This construct could also mediate the effect of past threat experiences (source of information) on the intentions of tourists to take preventive measures (protective motivation) [15] and, thus, can be included in threat appraisal.

Coping appraisal is the assessment of an individual’s ability to cope with threats, including self-efficacy, response efficacy, and response cost, after taking the adaptive response, which can characterize the individual’s beliefs about the coping response [11,38]. Response efficacy is the belief that taking the protective action will be effective in protecting the self or others. Self-efficacy is the perceived ability of the person to actually carry out the protective action. Response cost is the perception of any costs associated with taking the protective action [23]. In coping appraisal, response efficacy and self-efficacy both have positive effects on self-protective behaviors, whereas adaptive response costs decrease the likelihood of engagement [25].

These two cognitive appraisals both result in adaptive or maladaptive coping modes [26], for example, in mask-wearing behaviors [39] and stay-at-home behaviors [40]. Moreover, coping appraisal is better than threat appraisal in predicting tourist behaviors [41]. The relationship between these two appraisals has been verified, and threat appraisal has a positive and significant influence on coping appraisal [17]. Based on the relationship between cognitive appraisals and tourists’ protective behavioral intentions, the following hypotheses are proposed:

**Hypothesis** **4** **(H4).** *Tourists’ threat appraisals have positive effects on tourists’ coping appraisals.*

**Hypothesis** **5** **(H5).** *Tourists’ threat appraisals have positive effects on tourists’ social distancing intentions.*

**Hypothesis** **6** **(H6).** *Tourists’ coping appraisals have positive effects on tourists’ social distancing intentions.*

**Hypothesis** **7** **(H7).** *Both threat appraisal and coping appraisal act as mediators between perceived destination support and tourists’ social distancing intentions.*

### 2.3. Moderating Influence of Social Norms

Social norms are mainly studied in the field of online behaviors [42,43]. It refers to the individual’s perceptions about how others (e.g., friends, social media) behave or think a person ought to behave. Considering its importance as a social environmental factor, some studies suggested social norms as a direct antecedent of behavioral intention [44,45]. However, as they are generated from interpersonal interactions, social norms can serve as a moderator between perception and behavior as a kind of social influence, which may explain the inconsistent results from research that used social norms as an antecedent [43]. Some researchers proposed social norms as a potential source of heterogeneity in PMT studies [46]. Social norms moderate threat appraisal, whereas they could outweigh perceived risks and lead users to adopt computer services [47]. Social norms also moderate coping appraisal, and the effect of coping self-efficacy varied with the perception of social norms; higher perceived self-efficacy only urges people who are aware of high-level social norms, but not those with low-level social norms, to take protective measures [43]. Thus, this factor was chosen as a possible moderator in this study. The following hypothesis is proposed:

**Hypothesis** **8** **(H8).** *The mediational effect will be conditional on social norms.*

The final conceptual model that was developed based on PMT is presented in Figure 1.

## 3. Research Methodology

### 3.1. Data Collection

Considering the special circumstances and quarantine requirement during the COVID-19 pandemic in China, this study used an online panel survey to collect data. A pilot survey was first conducted from 28 to 30 April 2020, with participants who had domestic travel experience after February 2020. There were 30 valid questionnaires collected online to check the appropriateness of the survey. After improving and revising the formal survey, we then officially administered the questionnaires after the May Day holiday (from 2 May to 7 May, 2020).

With fewer than 10 new confirmed cases a day, most areas in China were considered low-risk, and the Chinese CDC implemented some key policies on travel health during the May Day holiday on 30 April. This five-day holiday was the first vacation after the resumption of national transportation. According to the Ministry of Culture and Tourism of the People’s Republic of China, the total number of domestic tourists in the country exceeded 100 million during the May Day Holiday. With mixed feelings of nervousness and enjoyment, the period after the May Day Holiday was an appropriate time for respondents to answer the survey.

Participants were recruited through an online panel survey provided by Sojump (www.wjx.cn (accessed on 2 May 2020))—the largest professional survey company in China. To ensure a feasible sample size and the inclusion of tourists diverse in age and profession, 1065 Chinese participants were recruited through the survey company with a random sampling approach. Our target population comprised residents who had travel experience across the nation, only participants who answered “yes” to the screening question (i.e., have you traveled cross-district and overnight, cross-city, or cross-province from February to May 2020?) were included in this study. A total of 655 responses were returned (response rate = 61.5%). After eliminating fast responses (answer time less than 3 min) and pattern answers, 605 questionnaires were retained for analysis (valid response rate = 92.4%).

### 3.2. Measures

Aside from sociodemographic variables, all of the items for the constructs were adapted from relevant previous studies and modified to the Chinese context to ensure content validity. The respondents marked their responses on a five-point Likert scale (where 1 = totally disagree; 5 = totally agree). To measure perceived destination support, four items were validated and applied by Ruan and colleagues were selected [18]. When coping appraisal and threat appraisal were considered as the second-order construct [17], these two appraisals were used as the second-order reflective measure following the guidelines of Wang and colleagues [17], and each first-order factor could only have two indicators [48]. For the appraisal process in PMT, the measurement variables and scales from the study by Milne and colleagues have been widely adopted [3,29,49]. Thus, all items, except for the new sub-dimension overcrowding perception, were mainly adapted from the study by Milne and colleagues (i.e., severity perception, vulnerability perception, self-efficacy, response efficacy, response cost) [49], which was modified in order to relate to the pandemic and tourism (e.g., ‘If I caught COVID-19 while traveling I would die prematurely’). The new sub-dimension of overcrowding perception in threat appraisal was suggested by Lu and Wei [15]. Social distancing intention was measured with three questions referred to Milne and colleagues [49] and was modified with the social distancing behaviors proposed by Chen and colleagues (e.g., ‘I intend to stay at least 1.5 m from other people while traveling’) [7]. Three items for moderator social norms were validated and applied by Chou and Sun [43]. Appendix A provides the detailed list of the measurement items.

### 3.3. Data Analysis

Descriptive statistics were generated in SPSS 21.0 (IBM Corporation, Armonk, NY, USA). A confirmatory factor analysis (CFA) and structural equation modeling (SEM) were performed using AMOS 18.0 (IBM Corporation, Armonk, NY, USA). In the first step, AMOS 18.0 was employed to conduct a CFA for two second-order factors. A second-order model was used to validate the relationship with two distinct second-order factors, including threat and coping appraisal. A CFA was also applied to estimate the appropriateness of the measurement model for extended PMT, and to determine the internal consistency of items and their construct validity.

The second step tested hypotheses, and SEM was employed based on the maximum likelihood estimation method. As noted by Anderson and Gerbing, before structural paths were estimated to test hypothesized relationships between latent variables, the properties of the measurement model were assessed [50]. Several indices were employed to measure the fit of the model, including the comparative fit index (CFI) and the root mean square error of approximation (RMSEA). CFI, NFI, and IFI values greater than 0.90, and an RMSEA value less than 0.08, are indicative of a good fit [51].

Third, to examine the mediating effects of appraisals toward tourists’ protection motivation processes, the bootstrapping technique (5000 subsamples) and Mackinnon’s PRODCLIN2 [52] were both applied. As noted by Preacher and Hayes, bootstrapping is the most powerful and feasible method testing the existence of mediating effects under most conditions [53]. Mackinnon’s PRODCLIN2, as recommended by MacKinnon and colleagues, is specifically designed to demonstrate indirect effects [54]. The results of the bootstrapping and PRODCLIN2 techniques assume that if the 95% confidence interval do not include zero, the mediating effect can be considered significant at the 0.05 level.

Last, the moderating effect (H8), which addressed whether the level of appraisals would vary according to the interaction effect of social norm, was investigated through a multi-group approach. To round values to the nearest integer, the sample was divided into three groups according to the mean value of scale items used to measure an individual’s social norm: low (1–2.4), medium (2.5–3.4), and high (3.5–5.0). The multi-group analysis method can be used to verify whether the same path model can be applied across different datasets. That is, if there is any existence of significant differences in the proposed structural model, then there is a stronger causal relationship between the paths (or weaker) for a high social norm group (or medium and low social norm group) as compared to their counterparts.

## 4. Results

### 4.1. Demographic Profile of the Sample

Table 1 shows the demographic characteristics of the respondents. The relatively higher response rate of college students might be related to the fact that most of the classes for college students were conducted online during the whole spring semester in China. This gives them more opportunities to travel during the pandemic period.

### 4.2. A CFA of PMT

We tested the model with AMOS 18.0. As a second-order model was used, we utilized a CFA to compare the first- and second-order models. The data defined three factors (severity perception, vulnerability perception, and overcrowding perception) of threat appraisal and three factors (self-efficacy, response efficacy, response cost) of coping appraisal.

First, we tested the model fit considering threat appraisal and coping appraisal as first-order models separately. Then, we tested the model fit considering these two constructs as second-order reflective constructs. Table 2 shows the results and the overall goodness-of-fit indices for the first- and second-order factor models, which provides a good fit to the data [50]. The results indicate that both threat appraisal and coping appraisal fit better as a second-order factor, and the new construct overcrowding perception is verified by the second-order CFA.

### 4.3. The Validity and Reliability of Measurement Variables

The CFA method was performed using AMOS 18.0 to estimate the validation of the scale and the internal consistency of items before analyzing the conceptual model. The results reveal a satisfactory fit to the data and are summarized in Table 3 [50,55]: χ^2^/dƒ = 2.635, (*p* < 0.001); NFI (0.946); GFI (0.940); IFI (0.966); CFI (0.965); RMSEA (0.052). As recommended, the composite reliability (CR)—the internal consistency of multiple indicators for each construct)—and average variance extracted (AVE)—the discriminant validity of major constructs—values met the minimum cut-off of 0.70 and 0.50, respectively [51]. The CR of the constructs ranged from 0.73 to 0.91 and most of the AVE values ranged from 0.52 to 0.72. Here, threat appraisal (0.49) as a second-order construct was marginally acceptable [56], and others all exceeded the recommended threshold of 0.50 [57].

### 4.4. Hypothesis Testing

SEM was employed based on bootstrap maximum likelihood estimation to test the full structural model and verify the hypothesized relationships between PDS, threat appraisal, coping appraisal, and SDI (Appendix A.). The overall goodness-of-fit indices were χ^2^/dƒ = 2.859 (*p* < 0.001); NFI (0.934); GFI (0.930); CFI (0.956); IFI (0.956); RMSEA (0.055), indicating acceptable structural model fit [51]. The results supported hypotheses 1–6 of the conceptual model. Specifically, PDS had significant positive effects on threat appraisal (0.14, *p* < 0.01), coping appraisal (0.41, *p* < 0.001), and SDI (0.20, *p* < 0.001). Threat appraisal had an extremely significant positive effect on coping appraisal (0.45, *p* < 0.001) and SDI (0.27, *p* < 0.001. Coping appraisal also had an extremely significant positive effect on SDI (0.34, *p* < 0.001).

### 4.5. Tests of the Mediating Effects of Appraisals

The bootstrapping technique and MacKinnon’s PRODCLIN2 were applied to examine the mediating effects of two appraisals toward tourists’ protection motivation processes. As shown in Table 4, the lower and upper 95% confidence intervals highlighting values of the path from PDS to SDI do not include zero; thus, the mediating effects of threat appraisal and coping appraisal were considered significant at the 0.05 level [53,54]. To explore the whole mediating process, MacKinnon’s PRODCLIN2 was applied [54]. The results indicated that threat appraisal and coping appraisal toward tourists’ social distancing behaviors partially mediate the relationship between PDS and SDI. The mediating process, first through threat appraisal then through coping appraisal, and the mediating effect of coping appraisal, was higher than that of threat appraisal. This finding supported H7.

### 4.6. The Moderating Role of Social Norms

A multi-group approach was used to investigate the moderating effect, which addressed whether the mediational effect was intervened by social norms. According to the mean value for the four items of social norms, the sample was divided into two groups [56]: 88 cases with low-level social norms (1–2.5) and 517 cases with high-level social norms (2.6–5.0). The free parameter estimates (χ^2^ = 701.54, dƒ = 316) was used to test the structural model, and a model with an equality constraint (χ^2^ = 731.83, dƒ = 328) was tested concurrently. The comparison of dƒ and χ^2^ (Δχ^2^ = 30.29, Δdƒ = 12) was significant (*p* = 0.003). This result revealed the moderating effect of social norms [56].

We employed AMOS 18.0 to test pairwise parameter comparisons, which can indicate if there are any significant differences between path coefficients among different social norms groups [55]. This comparison produced a Z-score indicating the statistical difference between groups on a specific path coefficient [58]. The results of the pairwise parameter comparisons are presented in Table 5. Regarding the mediating effect of threat appraisal on relationships between perceptual destination support and SDI, the effect of PDS on threat appraisal was non-significant in the high-level group. For the group with low social norms, the effect of threat appraisal on SDI was also non-significant. Regarding the mediating effect of coping appraisal on relationships between perceptual destination support and SDI, the path coefficients of PDS to coping appraisal for the ‘low-level social norms’ group (0.70) were significantly stronger than the corresponding path coefficients for the high-level groups (0.38) (Z = 2.51, *p* < 0.05). For the causal relationship between threat appraisal and coping appraisal, the former only had a significant positive effect on the latter in the high-level groups. As all mediation paths statistically differentiated low-level from high-level social norms except coping appraisal → SDI, the results partially supported H8.

## 5. Discussion and Implications

This study has four major empirical results. We empirically tested a second-order factor model of PMT; we examined the causal relationships among tourists’ PDS, coping appraisal, threat appraisal, and intentions to engage in social distancing behaviors; we examined the mediating role of two appraisals; and we examined the moderating effect of individual social norms on the whole model.

The results of a second-order factor analysis suggest that coping appraisal may be operationalized as a second-order factor including self-efficacy, response efficacy, and response cost. Threat appraisal was also operationalized as a second-order factor including severity perception, vulnerability perception, and overcrowding perception. There were significant relationships among PDS, coping appraisal, threat appraisal, and SDI. In addition, threat appraisal and coping appraisal toward the COVID-19 pandemic partially mediated the relationship between PDS and SDI, and these mediational effects were intervened by social norms.

### 5.1. Theoretical Implications

Given the scarcity of public health crisis research in tourism [20,59], the results of our study have important theoretical implications for both the tourism and public health literature. First, several tourism studies considered PMT in explaining tourists’ cognitive processes, and some destination factors as the sources of information, such as perceived destination risks and benefits [11,14,17,32]. However, very few have added perceived destination measures into this process, especially during pandemics. The positive role of PDS extends the protection motivation model during the pandemic period.

Second, the structural relationships between PDS, coping appraisal, threat appraisal, and SDI were tested. This ensured destination social distancing measures would increase the protective intentions of tourists, as the presence of destination support increased their levels of perceived threats and coping confidence. A growing amount of literature highlights that people may employ coping strategies to address negative emotions caused by public crises [60,61]. The results support previous research by verifying the second-order structure of PMT and the causal relationship between threat appraisal and coping appraisal, which extended the theory in a pandemic context. Additionally, consistent with previous studies on public protective intention induced by coping appraisal and threat appraisal [17,23], the results also offered important insights into destination authority and its positive effects on the cognitive appraisal of tourists. Most Chinese tourists who traveled during the pandemic were encouraged by the destination’s protective policies, despite the considerable limitations to individual freedom. They were willing to guide their behaviors with principles that were set by the government during the pandemic period.

Third, the moderated mediation model was examined. According to the results, the mediational effects of coping appraisal and threat appraisal are conditional on social norms. This is consistent with the findings of Chou and Sun [43], who suggested that social norms could serve as a moderator between perception and behavior instead of as an antecedent. Among tourists with high-level social norms, the implementation of the destination protective policy supports will not bring a threat. It will instead strengthen tourists’ coping confidence in protective behaviors, which have a significant positive effect on tourists’ SDIs. While the implementation of destination social distancing measures will bring a certain threat among tourists with low-level social norms, it will greatly enhance people’s coping confidence in themselves and society. Threat appraisal cannot strengthen tourists’ willingness to engage in social distancing behaviors among tourists within low-level social norms, but coping confidence has a positive effect on it. Thus, social distancing policy supports in tourist destinations can also enhance tourists’ SDIs, among tourists within low-level social norms. The higher the social norms, the higher the effectiveness of these measures. The results extend the previous work in this area by investigating the role of social norms in the relationship between perceived threat and protective intention. Given previous findings of divergence between people’s protective motivations and their protective travel behaviors (i.e., travel avoidance and cautions travel) during a pandemic [3,20,62], this study provides a new group perspective.

### 5.2. Managerial Implications

The tourism industry is a type of service industry where personal interaction is inevitable and, thus, policies and support from government and tourism companies has greatly affection. Considering the public’s fear caused by the COVID-19 outbreak, it is important to adopt effective protective strategies to improve the safety and coping confidence of tourists, during the pandemic or in a post-pandemic context. As China is recovering from the pandemic, the findings of this work have broader appeal.

First, the research suggests a need to explore tourists’ health protective behaviors while traveling during the pandemic period, and the results provide critical insight into the important role of PDS, which can encourage tourists to practice health protective behaviors. More concretely, destination practitioners and governments should provide stricter social distancing policies in the post-pandemic period to demonstrate that the tourism companies can control social distancing effectively, including real-time supervisory network and online services for tourists. These measures can strengthen the threat appraisal and coping appraisal of tourists toward COVID-19, and as a result, tourists’ intentions to engage in social distancing behavior while traveling can be enhanced.

Second, our findings indicate that tourists’ threat appraisals and coping appraisals bridge the relationship between PDS and SDI, and the mediating effect of coping appraisal is much greater than threat appraisal. This means that coping appraisal is useful in influencing tourists’ SDIs, and managers should focus on public confidence in coping with the virus during the COVID-19 pandemic.

Third, by testing the moderating effect of tourists’ social norms, the study offers new insights into how those with high-level social norms strengthen the effect of PDS on SDI with the mediation process of two appraisals. Among the ‘low-level social norms’ group, although the prevention and control policies of social distancing will affect the threat appraisal of tourists to a certain extent, it can greatly enhance coping appraisal and SDI, which can reduce the spread of the virus and effectively suppress the pandemic in the area. Among the ‘high-level social norms’ group, prevention and control policies will not affect the threat appraisal of tourists, but will only enhance the confidence of tourist, in that the destination has handled the public health crisis properly. Therefore, the restriction of prevention and control policies will not affect the quality of travel experience.

Managers should consider the trade-offs between health protective restrictions on tourists and the threats to tourist destinations in the ‘low-level social norms’ group. Although destination measures have an effective positive influence on the social distancing behaviors of tourists, they can also increase panic in tourists in tourist destinations. However, managers in the ‘high-level social norms’ group do not have to worry about threats to tourist destination images as a result of social distancing restrictions. Considering the political cultural background of different countries, these results can be better practiced at the national level. In China, most tourists are classified into the ‘high-level social norms’ group. Tourists have much more travel confidence and social responsibility with the comprehensive protection measures released by the destination government. This is evidenced by the fact that the total number of domestic tourists during the Chinese May Labor Day holiday exceeded 100 million, despite the pandemic [63].

## 6. Conclusions

Considering how prevention and control policies can improve the risk assessment of tourists during a specific period, generate panic, and have adverse effects on the destination, destination managers must avoid comprehensive protective supports. However, this study finds that support measures involving social distancing in tourist destinations can effectively enhance the intentions of tourists to protect themselves in low-level and high-level social norm groups. In the ‘high-level social norms’ group, PDS has no significant effect on the threat appraisal of tourists. The results provide universal suggestions for destinations with different social backgrounds worldwide, during different public health crises.

Despite important contributions, there are several limitations of this study. Our research only considers the travel situation under the COVID-19 pandemic. The results can be generalized to other public health events, but not to all travel situations. In particular, the results only show the experiences of Chinese tourists. Future research should also reveal the antecedent and moderating roles of cross-culture differences (e.g., culture, ethnicity, and ruling parties) in the PMT process during the travel period. Furthermore, social distancing behavior is a typical protective method during the COVID-19 pandemic; thus, future research should pay attention to other protective behaviors in response to different public health diseases.

## Figures and Tables

**Table 1:** Characteristics of the sample (*N* = 605).

Characteristics	Frequency	Percentage	Characteristics	Frequency	Percentage
Sex			Education		
Male	258	42.64%	Primary school	3	0.50%
Female	347	57.36%	High school	46	7.60%
Age (years)			Junior college and undergraduate	397	65.62%
<18	14	2.31%	Graduate	159	26.28%
18–25	295	48.76%	Income		
26–30	112	18.51%	≤CNY 3500	340	56.20%
31–40	71	11.74%	CNY 3501–5000	81	13.39%
41–50	53	8.76%	CNY 5001–8000	65	10.74%
51–60	55	9.09%	CNY 8001–12,500	63	10.41%
>60	5	0.83%	>CNY 12,500	56	9.26%

**Table 2:** A confirmatory factor analysis.

Model Fit	Criteria Value	First-Order Factor Models (Threat Appraisal)		Second-Order Factor Model ^c^ (Threat Appraisal)	First-Order Factor Models (Coping Appraisal)		Second-Order Factor Model ^f^ (Coping Appraisal)
		One-factor model ^a^	Three-factor model ^b^		One-factor model ^d^	Three-factor model ^e^	
GFI	>0.9	0.70	0.99	0.99	0.81	0.98	0.98
CFI	>0.9	0.67	1.00	1.00	0.65	0.99	0.99
NFI	>0.9	0.67	0.99	0.99	0.65	0.98	0.98
IFI	>0.9	0.67	1.00	1.00	0.66	0.99	0.99
RMSEA	<0.08	0.35	0.05	0.05	0.27	0.06	0.06
	-	771.40	15.57	15.57	725.29	38.25	38.25
dƒ	-	9	6	6	14	11	11
χ^2^/dƒ	<5.0	85.71	2.59	2.59	51.81	3.48	3.48

Note: ^a^ shows an integrated model including all threat appraisal items. ^b^ Was analyzed by dividing threat appraisal items into three factors (severity perception, vulnerability perception, overcrowding perception). ^c^ Included three factors for threat appraisal as latent variables. ^d^ Shows an integrated model including all coping appraisal items. ^e^ Was analyzed by dividing coping appraisal items into three factors (self-efficacy, response efficacy, response cost). ^f^ Included three factors for coping appraisal as latent variables.

**Table 3:** Reliability and validity of constructs.

	Non-Standardized Factor Load	Mean	SE	*t*	*p*	Standardized Factor Load	AVE	CR
PDS1	1	3.78				0.80	0.72	0.91
PDS2	1.06	3.75	0.04	23.87	***	0.86		
PDS3	1.12	3.74	0.04	25.24	***	0.90		
PDS4	1.07	3.80	0.05	23.39	***	0.84		
TA-SP	1	4.21				0.64	0.49	0.74
TA-PV	0.78	3.66	0.11	7.33	***	0.62		
TA-OP	1.11	3.83	0.12	9.64	***	0.82		
CA-SE	1	3.57				0.74	0.53	0.76
CA-RE	1.36	3.97	0.13	10.58	***	0.90		
CA-RC	–0.34	2.36	0.05	6.56	***	–0.47		
SDI1	1	4.22				0.78	0.70	0.88
SDI2	1.15	4.28	0.05	22.07	***	0.87		
SDI3	1.14	4.32	0.05	22.00	***	0.86		

Note: *** *p* < 0.001.

**Table 4:** The mediating effect of two appraisals using bootstrapping and Mackinnon’s PRODCLIN2 techniques.

Paths	Indirect Effects 95% CI (Bootstrapping)						MacKinnon’s PRODCLIN2 95% CI	
	Bias-Corrected			Percentile				
	Lower	Upper	*p*	Lower	Upper	*p*	Lower	Upper
PDS→SDI	0.098	0.255	***	0.096	0.253	***		
PDS→TA→SDI	-	-	-	-	-	-	0.01	0.07
PDS→CA→SDI	-	-	-	-	-	-	0.02	0.88
PDS→TA→CA	-	-	-	-	-	-	0.01	0.10
TA→CA→SDI	-	-	-	-	-	-	0.07	0.24

Note: *** *p* < 0.001.

**Table 5:** Pairwise parameter comparisons.

Paths	Z	*p*	Standardized Factor Load	
			Low Social Norms	High Social Norms
PDS→TA	2.80	0.005 **	0.37 ***	0.03
TA→SDI	2.33	0.020 *	−0.05	0.28 ***
PDS→CA	2.51	0.012 *	0.70 ***	0.38 ***
CA→SDI	−1.39	1.834	0.44 *	0.30 ***
TA→CA	3.93 ***	<0.001 ***	0.013	0.43 ***

Note: *** *p* < 0.001; ** *p* < 0.005; * *p* < 0.01.

**Figure A1:**
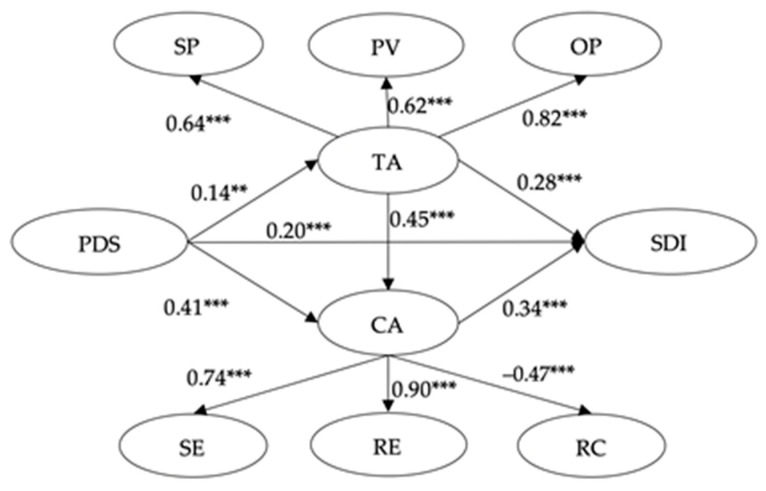
Structural model. (** *p* < 0.005, *** *p* < 0.001; standardized parameter estimates).

## Data Availability

All data used during the study were provided by a third party Sojump (www.wjx.cn, accessed on 2 May 2020)—the largest professional survey company in China. The raw/processed data required to reproduce these findings cannot be shared at this time as the data also forms part of an ongoing study.

## Appendix A

**Table A1:** Details of the constructs.

Constructs	Items
Perceived destination support (PDS)(4)	The destination had implemented control measures to prevent mass gatherings.
	The destination has built online services to prevent mass gatherings.
	The destination has implemented control measures to ensure tourists follow social distancing measures.
	The destination has built a supervisory network to provide tourists with information (to avoid getting COVID-19).
Threat appraisal (TA)	
Severity perception (SP)(2)	If I caught COVID-19 while traveling, I would suffer a lot of pain.
	If I caught COVID-19 while traveling, I would die prematurely.
Vulnerability perception (PV)(2)	My chances of catching COVID-19 while traveling are rather large.
	It is possible that I will catch COVID-19 while traveling.
Overcrowding perception (OP)(2)	Destination crowding may harm my satisfaction with the scenic spot.
	Destination crowding may harm the natural and human landscape.
Coping appraisal (CA)	
Self efficacy (SE)(2)	I am confident that I can prevent myself from catching COVID-19 by engaging in social distancing behavior.
	Engaging in social distancing behavior while traveling would be easy for me.
Response efficacy (RE)(2)	Engaging in social distancing behavior while traveling is a good way of reducing the risk of catching COVID-19.
	If I were to engage in social distancing behavior while traveling, I would lessen my chances of catching COVID-19.
Response cost (RC)(3)	Engaging in social distancing behavior while traveling would cause me too many problems.
	I would be discouraged from engaging in social distancing behavior while traveling as it would reduce the pleasure of travel.
	I would be discouraged from engaging in social distancing behavior while traveling because I would feel silly doing so.
Social distancing intention (SDI)(3)	I intend to stay at least 1.5 m from other people while traveling.
	I intend to avoid using public transport due to COVID-19 while traveling.
	I intend to avoid going to crowded places due to COVID-19 while traveling.
Social Norms (SN)(4)	I will engage in similar social distancing behaviors as my friends.
	I will engage in similar social distancing behaviors as fellow travelers.
	I will engage in social distancing behaviors in a way the way the government supports doing so.
	I will engage in social distancing behaviors in a way the way the scenic spot supports doing so.
